# Habitat alteration and fecal deposition by geese alter tundra invertebrate communities: Implications for diets of sympatric birds

**DOI:** 10.1371/journal.pone.0269938

**Published:** 2022-07-01

**Authors:** Scott A. Flemming, Paul A. Smith, Lisa V. Kennedy, Alexandra M. Anderson, Erica Nol

**Affiliations:** 1 Environmental and Life Sciences Graduate Program, Trent University, Peterborough, Canada; 2 Environment and Climate Change Canada, Pacific Wildlife Research Centre, Delta, Canada; 3 Environment and Climate Change Canada, National Wildlife Research Centre, Ottawa, Canada; 4 Migratory Bird Center, Smithsonian Conservation Biology Institute, Washington, DC, United States of America; 5 Department of Biology, Trent University, Peterborough, Canada; Universitat Autonoma de Barcelona, SPAIN

## Abstract

Over the last 60 years, Arctic goose populations have increased while many sympatric tundra nesting bird populations have declined. Hyperabundant geese have well-documented effects on tundra habitats, which can alter habitat use by sympatric bird species. These habitat changes may also alter invertebrate communities and abundances, with potentially important, but as of yet, undocumented effects on insectivorous birds such as shorebirds. Here, we determined the effects of goose-induced habitat alteration on invertebrate communities and relate the observed changes to shorebird diet. At sites and habitat types representing a gradient of goose influence, we identified goose-related changes in ground cover and linked these factors to variation in invertebrate communities. We then used DNA metabarcoding to characterize the diet of six shorebird species across sites and identify inter-site variation in abundance, biomass, and timing of emergence of dominant shorebird prey items. Invertebrate diversity and richness did not vary either among sites or habitat types. However, for prey items identified as part of the shorebird diet, we found significantly higher abundances and biomasses at a moderately goose-influenced site than at either low or high goose-influenced sites. Biomass of Tipulidae, the dominant prey taxon for shorebirds at the study sites, was 7.5 times higher at the moderately goose-influenced site compared to the site where goose influence was minor. We attribute this enhancement of prey biomass to both the fertilizing effect of goose fecal pellets and the moderate grazing pressure. Many studies have documented adverse effects of overabundant geese, but here we show that a moderate degree of goose grazing can lead to enhanced biomass of invertebrates, with the potential for improved shorebird foraging success and chick growth. These benefits, however, might be outweighed by negative effects of goose-induced habitat alteration and predation pressure.

## Introduction

Worldwide, many populations of Arctic-breeding shorebirds are declining [1, 2]. In North America, disproportionate declines are occurring in Eastern and Central Arctic regions [3–5]. While human-induced habitat alteration at non-breeding sites and climate change across their ranges are thought to be the greatest threats for many shorebirds [6, 7], large populations of light geese (Snow Geese, *Chen caerulescens* and Ross’ Geese, *Chen rossii*) breeding on or near shorebird breeding habitats could contribute to declines at local or regional scales [8, 9]. Light geese can increase the risk of nest predation for shorebirds by attracting and/or subsidizing generalist predator populations [10–12] and affect their nest site selection by altering habitat [13]. These habitat changes could also influence the diversity or abundance of arthropods, which in turn are prey for the many species of insectivorous birds, such as shorebirds, that breed sympatrically with geese. The nature and importance of these possible cascading effects of goose-induced habitat alteration on invertebrate communities, and thus shorebird prey availability, remain largely unknown.

Goose-induced habitat alteration could influence invertebrate communities in several ways. Changes in habitat complexity, such as vertical structure, and plant biomass through conversion of vegetated wet meadows to exposed sediment [14–16] result in lower abundance and diversity of herbivorous invertebrates or invertebrates that rely on vegetation for breeding and hunting [17, 18]. In coastal wetlands, elevated soil and pond salinity caused by increases in soil temperature and evapotranspiration from loss of vegetative cover [19] could make some habitats inhospitable for heat- and salt-sensitive species [8]. Goose-induced temporal changes in micro-habitat characteristics can also affect the timing and length of emergence for some species [20]. Alternatively, soils enriched through fecal deposition around goose colonies could enhance entire invertebrate communities or the abundances of just a few families [21–23].

Most tundra-breeding birds are insectivorous [24], and for these species, the abundance and diversity of invertebrates has a significant influence on shorebird adults and chicks [25–27]. The timing of chick hatch for insectivorous birds is thought to have evolved to coincide with timing of emergence in invertebrates, and phenological mismatch between these two events is a predicted consequence of climate change [28]. Although recent studies have found variable support for this hypothesis [26, 29–31], the abundance of key prey items is an important determinant of reproductive success, with demonstrated impacts on population dynamics of insectivorous birds [27, 32].

The effects of light geese on nesting shorebirds through top-down changes in predation pressure [10–12], and habitat availability, which, in turn, influence nest site selection [13], are well described. Here, we sought to identify any bottom-up pressures exerted indirectly by geese on insectivorous birds such as shorebirds, through changes in availability of invertebrate prey. Our objectives were to 1) confirm gradients of goose-induced habitat alteration were present at scales relevant to invertebrate communities, 2) examine any such effects of goose-induced habitat alteration on invertebrate communities, 3) determine which taxa of invertebrates are most frequently consumed by tundra-nesting shorebirds by characterizing their diet, and 4) identify any effects of geese on the biomass or timing of emergence of dominant shorebird prey items.

To address these objectives, we conducted research at three study sites situated at increasing distances from a light goose colony in the eastern Canadian Arctic. We predicted that invertebrate community diversity would be lowest at the site with the most pronounced goose-induced habitat alteration and that any goose-related effects on invertebrate diversity would depend on the extent of vegetation loss. We then characterized decline in diversity in response to a reduction in vegetative cover. We used DNA metabarcoding to identify the preferred diet items for shorebirds, and determined how biomass and timing of emergence of dominant prey items varied with distance from the goose colony. We then discuss the potential mechanisms for this variation in invertebrate diversity and abundance, and the consequences for shorebirds breeding in goose-altered environments.

## Methods

### Study sites

**Fig 1:**
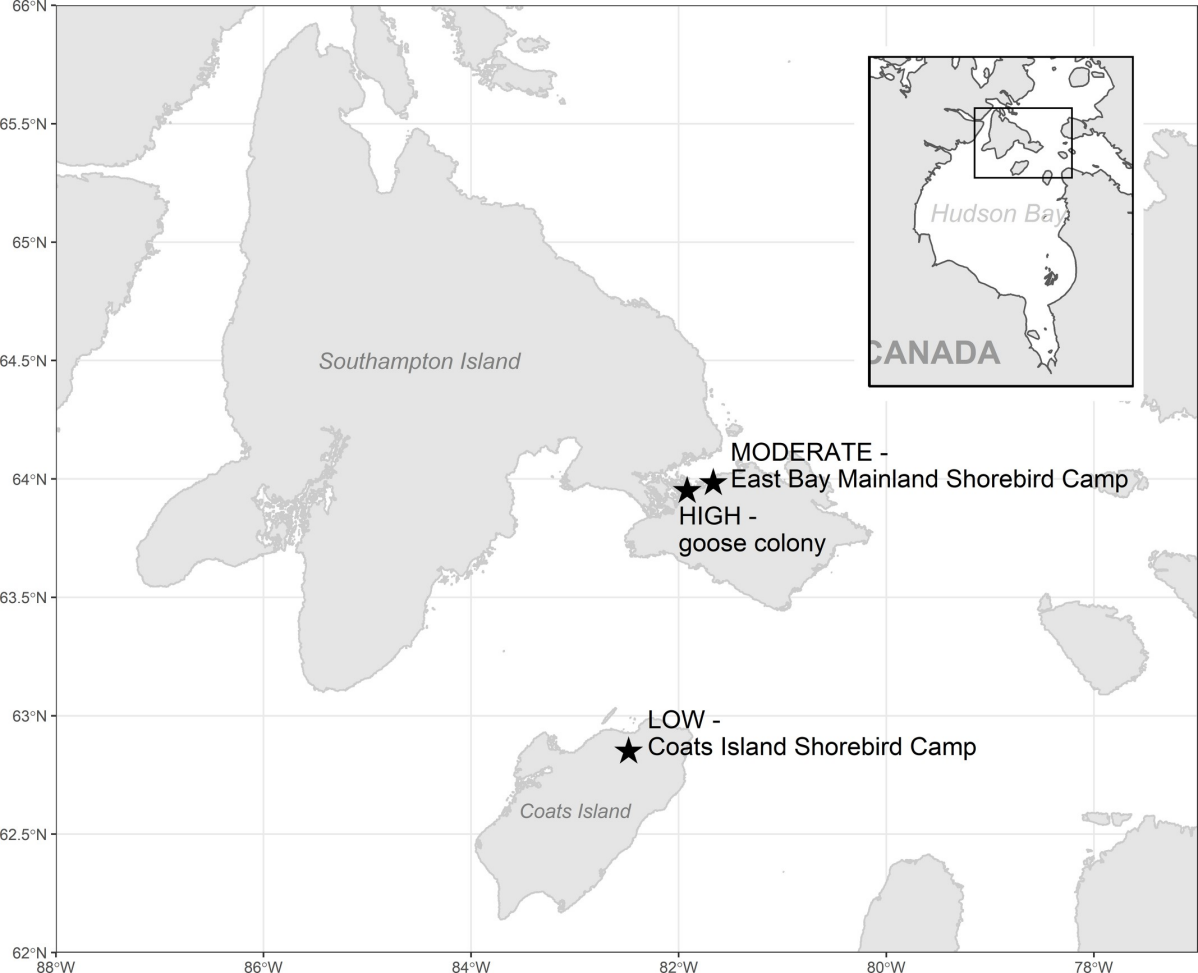
Locations of three study sites situated at increasing distances from a mixed lesser snow and Ross’ goose colony on Southampton Island and Coats Island, Nunavut.

All handling and sampling procedures were approved by Environment and Climate Change Canada’s Animal Care Committee and adhered to Federal and Territorial permits (e.g., NUN-SCI-14-05, WL2016-053). We examined the effects of geese on invertebrates at three sites with different distances from a large Lesser Snow Goose (*Chen caerulescens caerulescens*) colony in the Qaqsauqtuuq (East Bay) Migratory Bird Sanctuary on Southampton Island, Nunavut (Fig 1). The population of geese within this colony and an adjoining one have nearly doubled from a total of 156,700 breeding birds in 1997 to 289,700 in 2014 [33] (J. Leafloor, *unpublished*).

The first study site (6 km^2^) is situated within the goose colony. The second site, East Bay Mainland Shorebird Camp (12 km^2^) is situated within the sanctuary, ~10km to the east of the goose colony. Although East Bay Mainland is not typically used for breeding by light geese, family groups use the site later in the season for foraging. The third study site, the Coats Island Shorebird Camp (12 km^2^), is situated on Appatuurjuaq (Coats Island) ~135km south of East Bay Mainland. Light geese do not breed regularly at this study site but it is used for breeding by small numbers of Cackling Geese (*Branta hutchinsii*), and by staging light geese during migration in late July. Previous studies at these sites have confirmed a gradient of goose effects at larger scales [12, 13], thus, we refer to them here as high (goose colony), moderate (East Bay Mainland), and low (Coats Island) goose influence sites. We carried out fieldwork at the three sites throughout the shorebird breeding season in June and July of 2015–2017.

### Habitat characteristics

Because invertebrate communities differ among habitats we conducted habitat surveys near each of the pitfall traps (five traps per habitat type per year) within each of the three study sites. In 2015 and 2016, at each location we identified the dominant broad habitat type (lowland habitats: sedge meadow, scrub willow, and moss carpet; upland habitats: dry heath, gravel ridge, and intertidal), as described by [34] within a 75m^2^ area surrounding the random point. We then estimated the proportional cover of ground cover types (rock, *Dryas sp*., lichen, willow, moss, and graminoid), and counted the number of goose fecal pellets (fresh and old) within a 1m^2^ circle surrounding the random point (see [13]).

### Invertebrate communities

To characterize invertebrate communities at each study site, we collected invertebrates with pitfall traps placed randomly within each of the six habitat types (five traps per habitat type). The pitfall traps consisted of yellow plastic cups, with a mouth diameter of 14cm, dug into the ground so that the lip was even with the soil. We then filled each trap with approximately 5cm of diluted propylene glycol and two drops of triton soap to break the surface tension. We reset each pitfall trap about every seven days to total four collection periods throughout the peak of the shorebird breeding season (24 June– 24 July) except when the schedule was interrupted due to inclement weather or the presence of polar bears (*Ursus maritimus*). To standardize abundance measures we calculated the mean number of invertebrates per day per trap, for each family or other taxonomic groupings. In the field, we drained the traps of all liquids and stored samples in Falcon tubes with 95% ethanol. In the lab, each sample was sorted to family, where possible, using [35]. We also measured the lengths of a subsample of individual invertebrates per site (up to 20), for each family that we identified as shorebird diet items (see below). Using these measurements, we estimated biomass per sampling period using length-mass equations for invertebrate orders and families developed by [36]. When calculating site-specific biomass we multiplied the biomass of invertebrates at each study site by the proportional availability of each habitat type as described by [13].

### Shorebird diet

We characterized diets from six tundra-nesting shorebird species that consistently breed at all three sites: Dunlin (*Calidris alpina*), Semipalmated Sandpiper (*Calidris pusilla)*, White-rumped Sandpiper (*Calidris fuscicollis*), Red Phalarope (*Phalaropus fulicarius*), Black-bellied Plover (*Pluvialis squatarola*), and Ruddy Turnstone (*Arenaria interpres*). For diet characterization we used DNA metabarcoding, a technique that has proven successful in identifying diets of hummingbirds [37], waterthrushes [38], and swallows [39], but less attention has been paid to its use on shorebirds [40]. We applied this technique to shorebird fecal samples to characterize diet because the method is non-invasive, requires less expertise in identification, and provides a higher taxonomic resolution than observational or gut contents analyses [41]. DNA metabarcoding uses high-throughput sequencing, and matches the identified sequences to a reference database from known species.

To collect shorebird fecal samples, we captured individual shorebirds on their nests throughout the breeding season using bownet traps. We placed each bird in a covered sterilized plastic holding container lined with wax paper for a maximum of five minutes. We released the bird after it defecated and transferred the fecal sample to a microcentrifuge tube using a sterilized plastic spoon. We then stored samples at -20 degrees Celsius in 95% ethanol. After each individual capture, we sterilized the holding container with 70% ethanol and replaced the waxed paper. All fecal samples were processed at the Canadian Centre for DNA Barcoding (CCDB) at the University of Guelph, Guelph, Ontario, Canada, following protocols outlined by [37] and [42]. We used primers designed for arthropods ([43]; with a 157-bp section of the COI barcode region amplified), annelids (161-bp), microalgae (168 -bp), mollusks (161-bp), and amphipods (193-bp). All handling and sampling procedures were approved by Environment and Climate Change Canada’s Animal Care Committee and adhered to Federal and Territorial permits (e.g., NUN-SCI-14-05, WL2016-053).

### Statistical methods

#### Habitat characteristics and invertebrate communities

We used MANOVA to identify differences in proportional ground cover and fecal pellet abundance among study sites, habitat types, and a study site by habitat interaction. To identify differences in invertebrate communities among study sites and habitat types we used PERMANOVA. We included site, habitat type, and a site by habitat interaction as predictors. We then used MANOVA to identify differences in the abundances (log+1 transformed) of the top five most abundant invertebrate families across sites, total diversity (Shannon-Weiner Index), and species richness using site, habitat type, and a site by habitat type interaction as predictors.

To link invertebrate communities with habitat characteristics, we analysed habitat survey data stratified by dominant habitat type with pitfall traps. We then used a Redundancy Analysis (RDA) to identify relationships between continuous environmental variables (goose fecal pellet count, and proportions of rock, *Dryas*, lichen, moss, willow, and graminoid) and abundance at the family level. To assess the strength of the relationship between environmental variables and invertebrate abundance, we used the significance of the canonical axes and a Monte Carlo test with 999 permutations.

#### Shorebird diet and prey availability

Shorebird captures were time consuming and nest abundances varied significantly among study sites, which affected our rate of sample collection. Thus, we had too few samples to analyse differences in shorebird diet across the three sites (Black-bellied Plover n = 8; Dunlin n = 6; Red Phalarope n = 23; Ruddy Turnstone n = 9; Semipalmated Sandpiper n = 10; White-rumped Sandpiper n = 24). Instead, we identified the relative use of invertebrate taxa as shorebird prey by calculating the frequency of occurrence of each invertebrate family in each shorebird species’ diet across the three study sites combined. Based on visual inspection of frequency of occurrence, we classified the top five prey items as ‘important’ and conducted more detailed analyses of their biomass and timing of emergence.

To identify differences in prey biomass from the pitfall traps we used a generalized linear model with invertebrate family and site, and an interaction between the two as predictors. For an analysis of emergence timing, we used a generalized additive model with abundance (per collection period, log-transformed) as the response, and study site, prey family, collection period, year, and an interaction between all four as the predictors. All statistical analyses were performed in R Version 3.6.1 [44].

## Results

### Habitat characteristics

Habitat characteristics varied by study site (Wilks_2, 280_: 0.72449, p < 0.001), habitat type (Wilks_5, 280_: 0.10603, p < 0.001), and an interaction between the two (Wilks_10, 280_: 0.66762, p < 0.001; S1 Table). Fecal pellet counts were lower at the low goose influence site than the other two sites (F_2, 280_: 9.98, p < 0.001), and highest in moss carpet habitat and lowest in gravel ridge (F_5, 280_: 5.59, p < 0.001). Rock cover was highest at the moderate goose influence site (F_2, 280_: 4.66, p < 0.05) and in the gravel ridge habitat type (F_5, 280_: 63.00, p < 0.001), and lowest at the low goose influence site and in sedge meadow. Lichen (F_5, 280_: 23.21, p < 0.001) and *Dryas sp*. coverage (F_5, 280_: 34.06, p < 0.001) were both highest in dry heath, but lowest in moss carpet and intertidal, respectively. As expected, moss cover was highest in moss carpet and lowest in gravel ridge (F_5, 280_: 32.12, p < 0.001). Willow cover varied by a site and habitat type interaction (F_10, 280_: 2.70, p < 0.01). Among study sites (F_2, 280_: 29.06, p < 0.001), graminoid cover was highest at the low goose site and lowest at the high goose site, particularly within sedge meadow habitat (F_5, 280_: 38.47, p < 0.001) while the reverse was true in intertidal habitat (F_10, 280_: 1.99, p < 0.05).

### Invertebrate abundances and taxon richness

Over two summers at three study sites we collected a total of 877,678 individual invertebrates comprising 36 taxa. Invertebrate communities differed among study sites (F_2, 161_ = 26.67, p < 0.001, R^2^ = 0.14), habitat types (F_5, 161_ = 22.25, p < 0.001, R^2^ = 0.28), with a study site by habitat type interaction (F_10, 161_ = 6.93, p < 0.001, R^2^ = 0.18). A large number (>700 000) of Collembolans were collected during one sampling period in one trap from the intertidal zone at the low goose influence site, comprising ~93% of all individuals collected during this study and skewing the proportions of all other invertebrates. Excluding Collembola, Sciaridae (27.55%), Linyphiidae (14.13%), Chironomidae (12.14%), Muscidae (9.67%), and Tipulidae (4.54%) comprised the top five invertebrate families (~70% of all individuals) across sites. Most invertebrate individuals captured were adults.

**Fig 2:**
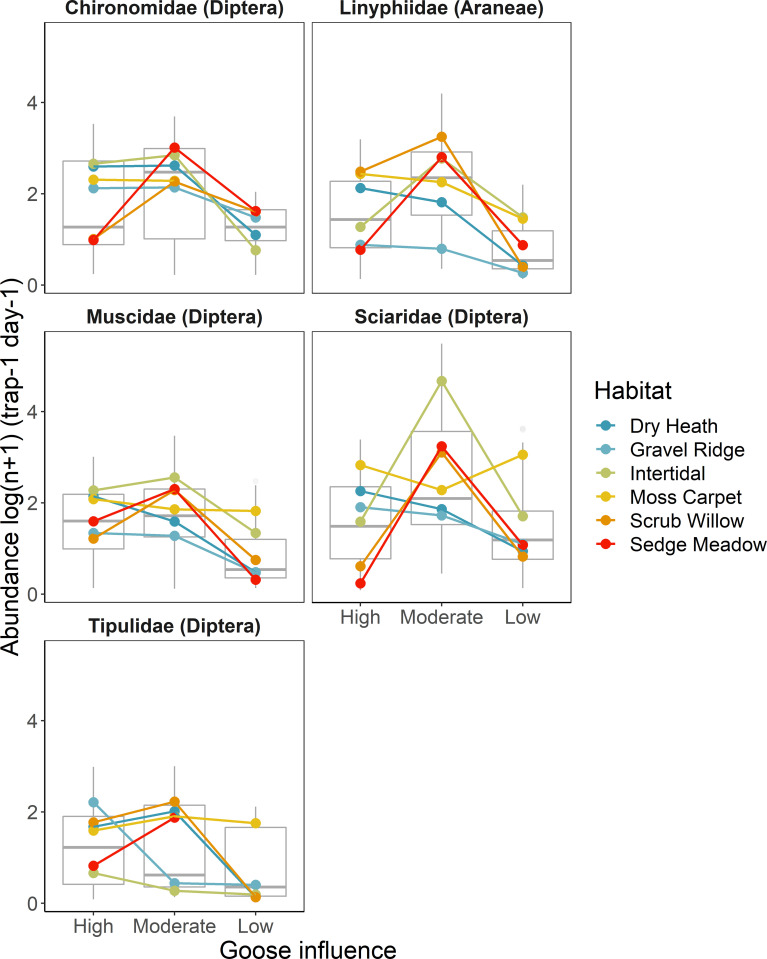
Abundance (per trap per day, log transformed) for the five most abundant invertebrate families collected using pitfall traps in six dominant habitat types at three sites representing a gradient of goose influence.

Abundances of the top five invertebrate families varied among sites (Wilks’_2, 162_: 0.23, p < 0.001), habitat types (Wilks’_5, 162_: 0.14, p < 0.001), and a site by habitat type interaction (Wilks’_10, 162_: 0.12, p < 0.001; Table 1). Tipulidae abundance was highest at the moderate goose site in dry heath, moss carpet, scrub willow, and sedge meadow and at the goose colony in gravel ridge and intertidal areas. Chironomidae abundance was consistently highest at the moderate goose site and lowest at the low goose site across habitat types, except for moss carpet where it was highest at the goose colony (Fig 2; S2 Table). Linyphiidae abundance was highest at the goose colony in dry heath, gravel ridge, and moss carpet, and at the moderate goose site in intertidal, scrub willow, and sedge meadow. Muscidae abundance was highest within the goose colony in dry heath, gravel ridge, and moss carpet, and at the moderate goose site in intertidal, scrub willow, and sedge meadow. Sciaridae abundance was highest at the goose colony in dry heath and gravel ridge, at the moderate goose site in intertidal, scrub willow, and sedge meadow, and at the low goose site in moss carpet.

**Table 1:** Results of post-hoc ANOVAs testing the effects of site, habitat type, and a site by habitat type interaction on the abundances of the dominant five invertebrate families collected in pitfall traps situated at a gradient of goose influence.

Family	Variable	D.F.	F-value	*P*
Chironomidae	Goose influence	2, 162	13.05	<0.001
	Habitat type	5, 162	3.38	<0.01
	Goose influence x Habitat type	10, 162	3.53	<0.001
Linyphiidae	Goose influence	2, 162	91.72	<0.001
	Habitat type	5, 162	28.23	<0.001
	Goose influence x Habitat type	10, 162	10.70	<0.001
Muscidae	Goose influence	2, 162	70.34	<0.001
	Habitat type	5, 162	15.08	<0.001
	Goose influence x Habitat type	10, 162	5.88	<0.001
Sciaridae	Goose influence	2, 162	49.08	<0.001
	Habitat type	5, 162	23.83	<0.001
	Goose influence x Habitat type	10, 162	15.40	<0.001
Tipulidae	Goose influence	2, 162	25.01	<0.001
	Habitat type	5, 162	6.66	<0.001
	Goose influence x Habitat type	10, 162	4.02	<0.001

Shannon-Wiener diversity also differed among study sites (F_2, 161_ = 17.99, p < 0.001), habitat types (F_5, 161_ = 11.02, p < 0.001), and a study site by habitat type interaction (F_10, 161_ = 3.07, p < 0.01; Table 2). Similarly, richness differed among study sites (F_2, 161_ = 19.31, p < 0.001), habitat types (F_5, 161_ = 13.05, p < 0.001), and a study site by habitat type interaction (F_10, 161_ = 3.58, p < 0.001; Table 2). Diversity in dry heath, gravel ridge, and moss carpet was lowest at the goose colony and highest at the low goose influence site. In the remaining habitat types, diversity was highest at the moderate goose site and lowest at the low goose site.

**Table 2:** Shannon diversity and taxa richness of invertebrates across traps and periods but among study sites representing a gradient of goose effects and habitat types (mean ± SE).

Index	Goose influence	Dry Heath	Gravel Ridge	Intertidal	Moss Carpet	Sedge Meadow	Scrub Willow
Shannon	high	1.99 ± 0.08	1.94 ± 0.06	1.91 ± 0.16	2.12 ± 0.05	1.86 ± 0.15	1.98 ± 0.07
	moderate	1.96 ± 0.10	1.79 ± 0.15	1.98 ± 0.09	2.05 ± 0.16	2.10 ± 0.12	2.09 ± 0.09
	low	1.86 ± 0.15	1.73 ± 0.12	1.82 ± 0.20	1.98 ± 0.14	1.89 ± 0.16	1.87 ± 0.15
Richness	high	13.60 ± 0.64	12.10 ± 0.43	11.80 ± 1.04	17.50 ± 0.58	10.80 ± 1.01	13.10 ± 0.50
	moderate	12.80 ± 0.84	9.30 ± 0.86	13.30 ± 0.73	15.56 ± 1.50	16.90 ± 1.23	16.50 ± 0.90
	low	10.80 ± 0.98	8.20 ± 0.63	10.10 ± 1.05	13.70 ± 1.15	11.30 ± 0.92	11.00 ± 0.97

### Habitat associations

The RDA revealed relationships between environmental variables and invertebrate communities (F_7, 172_: 1.91, p < 0.001). Axis 1 explained 39% of the variation in taxon-specific abundance and was driven by a gradient of low (negative scores) to high (positive scores) goose pellets and moss cover (F_1, 172_: 5.23, p < 0.001; Fig 2). Axis 2 explained 18% of variation and was driven primarily by a gradient of rock (positive scores) to graminoid and moss cover (negative scores; F_1, 172_: 2.45, p < 0.001).

**Fig 3:**
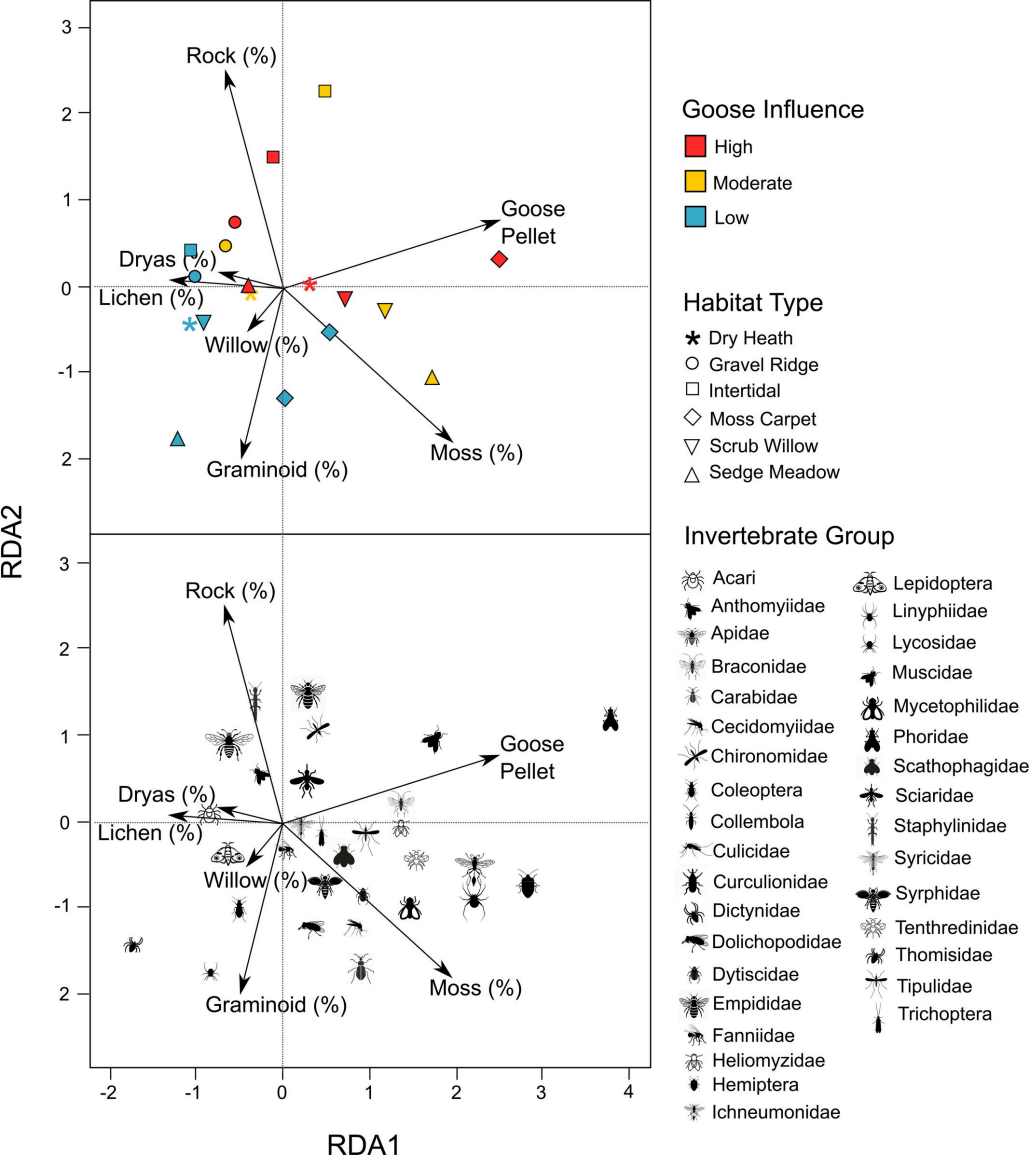
Ordination plots for redundancy analysis of invertebrate communities across study sites and habitat types. (top) Ordination showing the average RDA scores for three study sites with varying goose influence and six habitat types. (bottom) Ordination showing the effects of environmental variables on each invertebrate taxon. Goose pellets, moss cover, and graminoid cover were all significant environmental variables.

Of the seven environmental variables, moss (%; F_7, 172_: 3.21, p < 0.001; Fig 3), graminoid (%; F_7, 172_: 1.86, p < 0.05), and fecal pellet counts (F_7, 172_: 3.26, p < 0.001) were significant predictors of variation in invertebrate abundances. Hemiptera, Linyphiidae, and Ichneumonidae abundance were all positively associated with both fecal pellet counts and moss cover. Muscidae were positively associated with goose fecal pellet counts, Carabidae with moss cover, and Lycosidae with graminoid cover.

### Shorebird diet, prey availability, and biomass

We analysed a total of 82 fecal samples collected from 6 shorebird species, yielding 316,023 reads. This analysis identified 87 unique prey species from 23 families. Of the sequences from these samples, 92% were identified to the level of order and family, 85% to the level of genus, and 60% to species-level. Of the 93% of Operational Taxonomic Units (OTUs) that matched a reference sequence, 100% were identified to the order and family levels, 92% were identified to the genus-level and 65% identified to the species-level.

**Fig 4:**
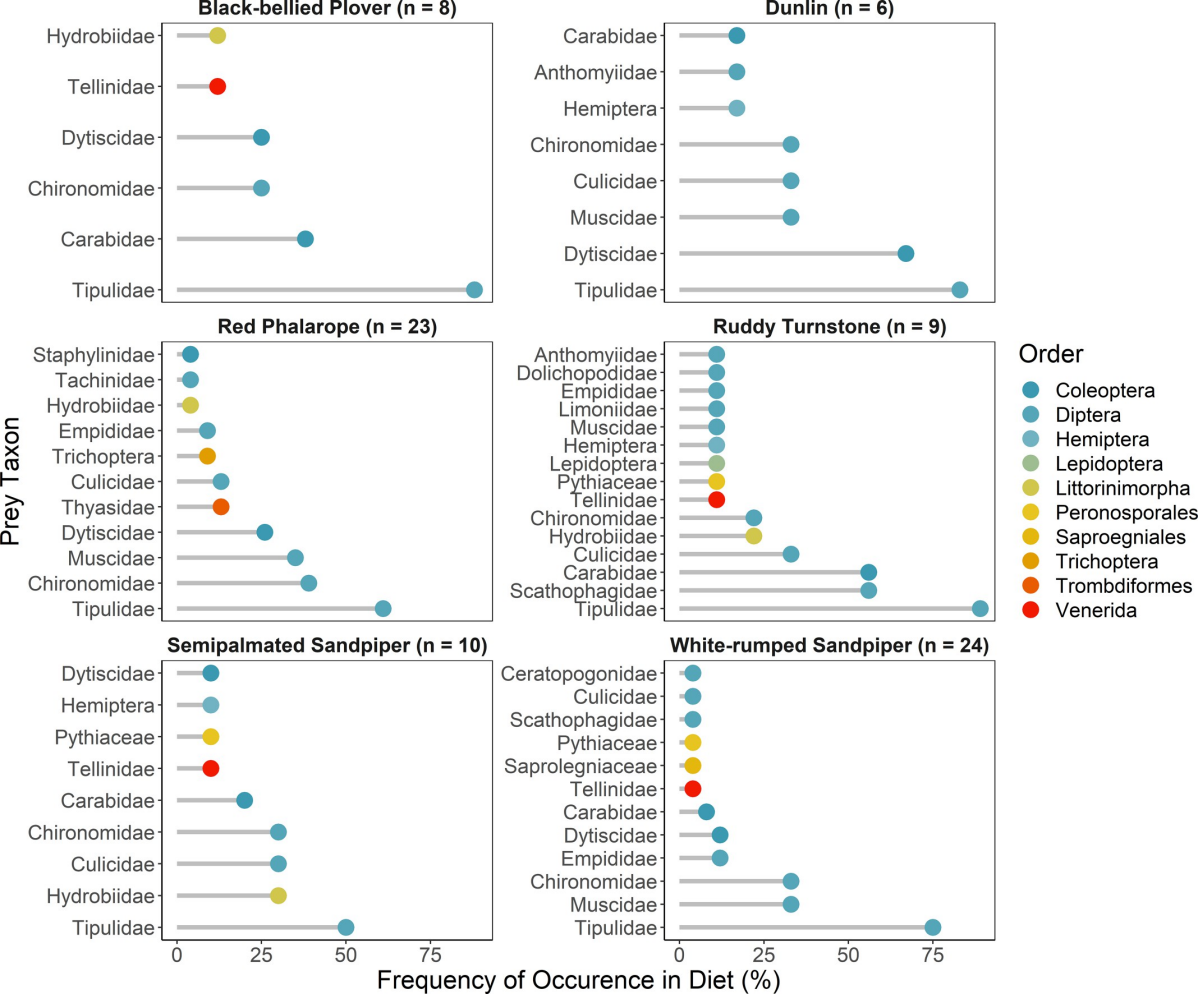
Frequency of occurrence of invertebrate families identified in fecal samples of each shorebird species using DNA metabarcoding.

Red Phalarope diets were the most diverse (34 taxa) while Black-bellied Plover diets were the least (14 taxa). This variation could be the result of variation in sample sizes among shorebird species and so should be interpreted with caution. Across all shorebird species Tipulidae (occurring in 72% of samples), Chironomidae (33%), Muscidae (22%), Dytiscidae (20%), and Carabidae (19%) were the most frequently occurring invertebrates in fecal samples. The frequency of prey occurrence also varied among shorebird species, but Tipulidae was present in the diet of all species (Fig 4).

**Fig 5:**
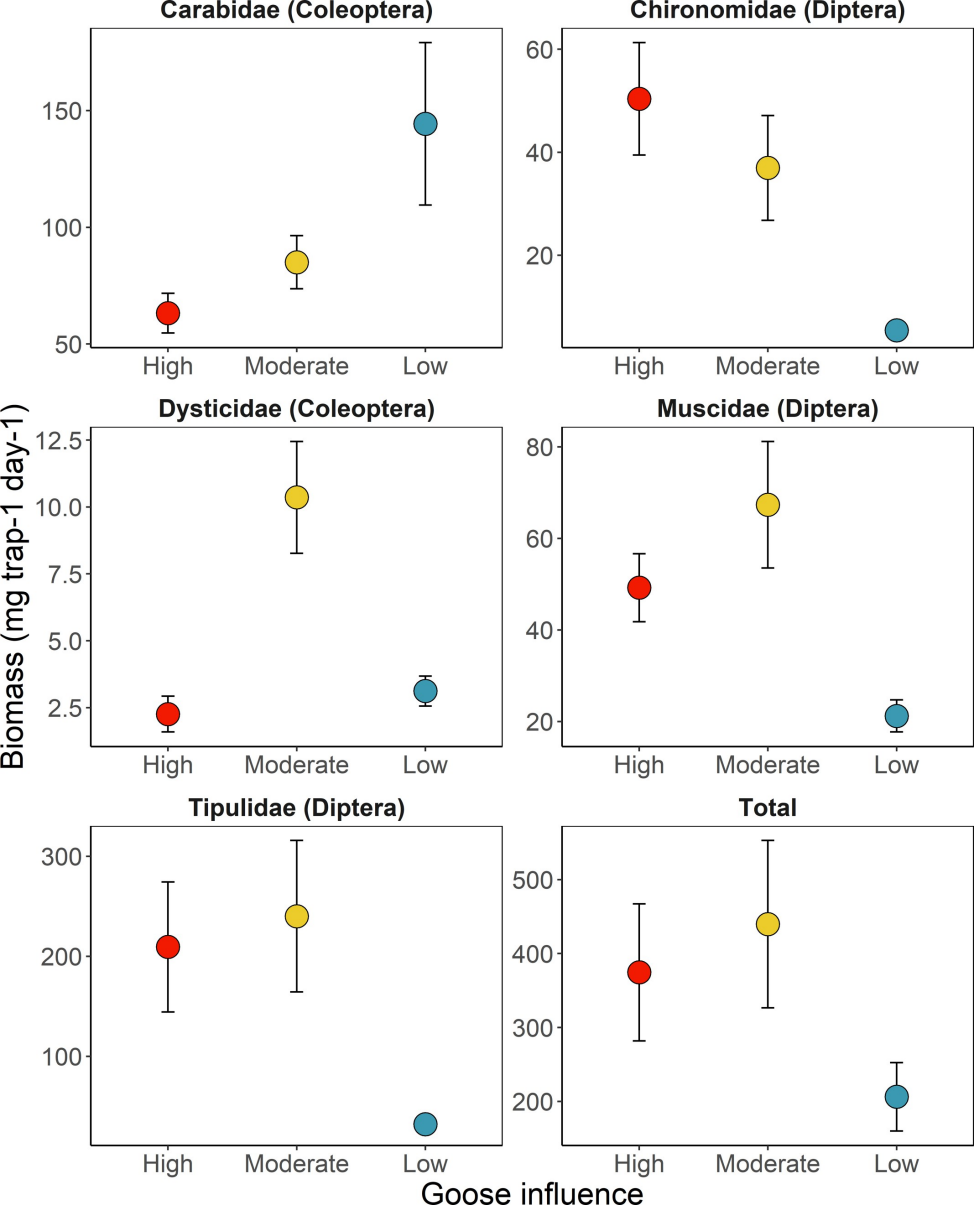
Estimated biomass (mean ± S.D.) corrected for proportional availability of habitat types of five dominant shorebird prey items among study sites representing a gradient of goose influence.

**Fig 6:**
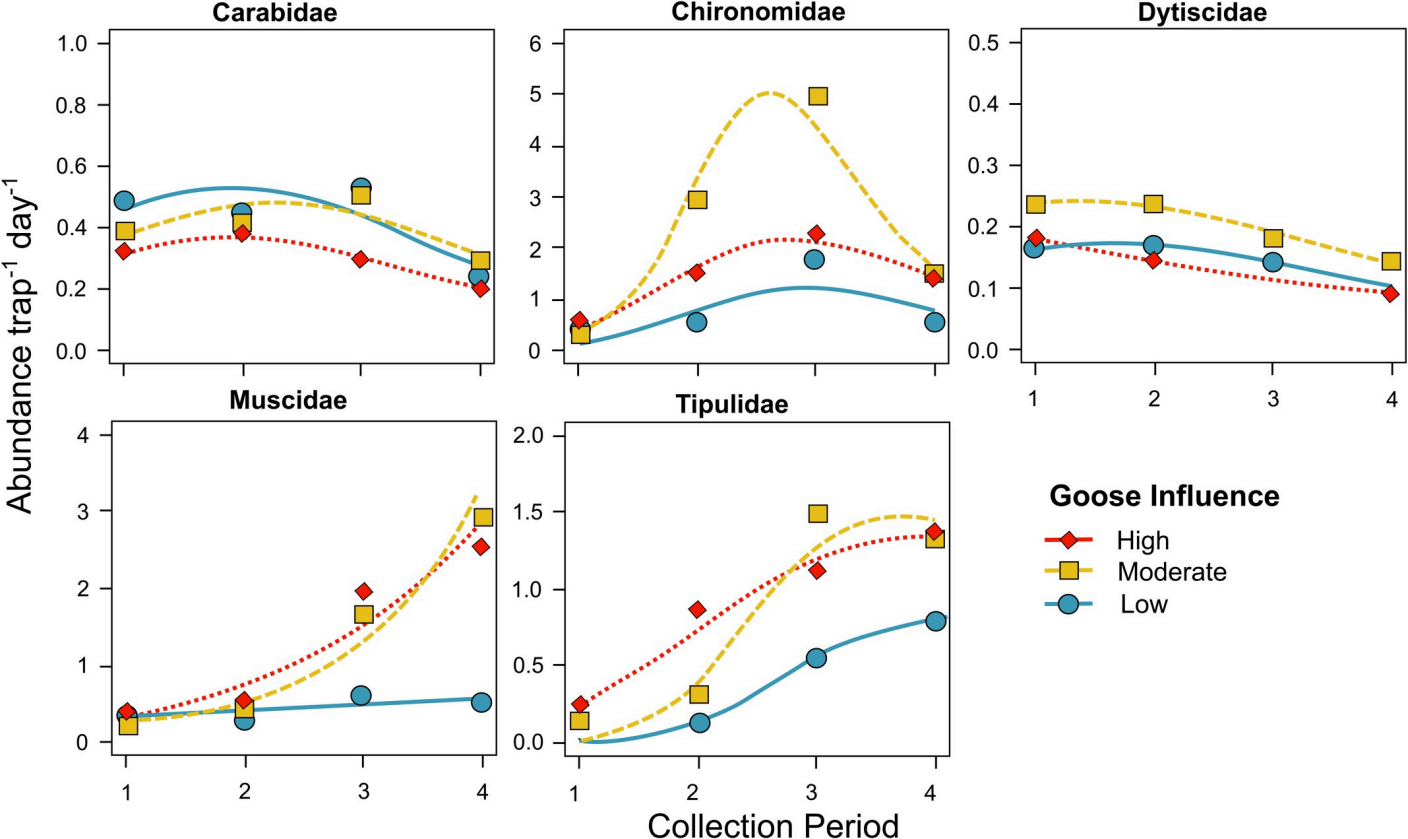
Abundance of dominant invertebrate prey items among study sites and sample periods along with emergence trends from GAMs. Sample collection period dates varied due to environmental factors but typically ranged from 23-Jun to 29-Jun (period 1), 29-Jun to 06-Jul (period 2), 06-Jul to 13-Jul (period 3), and 13-Jul to 21-Jul (period 4).

For these five primary shorebird prey families, the biomass captured in pitfall traps was greatest for Tipulidae (480.8mg trap^-1^day^-1^) and least for Dytiscidae (15.5; F_4, 128_: 44.13, p < 0.001; Fig 5). Total trap biomass of dominant prey items was greatest at the moderate goose site (437.8mg trap^-1^day^-1^) and least at the low goose site (198.7mg trap^-1^day^-1^; F_2, 128_: 8.61, p < 0.001). Biomass also varied by a taxon by site interaction (F_8, 128_: 3.42, p < 0.01). Prey item emergence over the four sampling periods varied among sites and families as indicated by three-way interaction between all three (F_16, 1341_: 2.00, p < 0.05; Fig 6). The four-way interaction including year was not significant (p > 0.05).

## Discussion

Our study identified effects of goose-induced habitat alteration on invertebrate communities, which could exert bottom-up pressures on shorebirds. However, contrary to our prediction, invertebrate biomass was higher at the site with moderate goose colony pressure, an effect which may benefit foraging adult shorebirds and their chicks.

During the incubation period, Arctic-breeding light geese are confined to foraging near their nests and within their breeding colony. Once goslings hatch, family groups may move well-beyond the colony creating a gradient in goose habitat effects [13]. At the study sites, goose pellet counts supported results from previous analyses at the sites [13], confirmed varying goose use, and further identified dominant habitat types used by geese. Compared to other habitat types, goose pellet counts and habitat indices varied less among study sites in dry heath and gravel ridge habitats; habitat types not frequently used by geese [13]. Conversely, measures of goose grazing and fecal pellet counts varied significantly among sites in sedge meadow and moss carpet habitats where higher proportions of their dominant forage, graminoids were found. At the high and moderate goose influenced sites, the sedge meadow habitat type also contained lower proportions of graminoids compared to at the low goose influence site, presumably because of grazing pressure [13]. These results are consistent with those of other studies describing goose-induced habitat alteration at multiple scales [45, 46] and have the potential to drive habitat-specific variation in invertebrate communities among sites.

We found invertebrate community composition differed at sites at different distances from the goose colony. Also, unexpectedly, invertebrate abundance was higher at the sites with high and moderate goose influence compared to the site with low goose influence. In lowland habitats (sedge meadow and scrub willow) invertebrate diversity and richness also tended to be highest at the moderately goose influenced site, while in upland habitats (dry heath and gravel ridge), diversity and richness were highest in the goose colony and lowest at where goose influence was low. These results are suggestive of multiple potential mechanisms of influence.

Fecal deposition by light geese has been suggested as a driver of changes in pond nitrogen and phosphorus levels [47–49] that alter food-web dynamics [50, 51] and may be responsible for the enhanced invertebrate biomass at the sites in and near the goose colony. We found that goose fecal pellet counts were a significant predictor of invertebrate abundances, and like patterns in goose pellets, invertebrate abundance was higher at the two study sites on Southampton Island (where geese are abundant). Similar results have been found on Svalbard Island, Norway, where fecal pellet counts can increase phytoplankton, invertebrate taxon richness, and alter species composition [51].

Although we did not measure pond or soil chemistry, fecal pellet densities at the sites in and near the goose colony were over six times higher than at Coats Island, where breeding geese are absent. Furthermore, across Southampton Island (where the goose colony is situated), [52] described a ~2.5-fold increase in total phosphorus of ponds over a 13-year period; this effect was most pronounced in ponds situated within goose colonies.

Fecal deposition at the goose colony could also be subsidizing Muscidae populations. Some Muscidae species may rely on fecal matter for food or laying eggs [53, 54], perhaps explaining our finding that the abundance of individuals within this fly family was nearly seven times higher at and near the goose colony than at the low goose site. The taxonomic resolution of our pitfall traps was, however, too coarse to identify whether some species within this family were favoured, and as such, this hypothesis requires further investigation.

Fecal deposition by geese only partially explains the variation in invertebrate communities. Invertebrate community composition and abundance are often driven by micro-climate characteristics such as temperature which can vary by vegetation type and coverage [55–58]. At the two lowland habitat types (sedge meadow and moss carpet), invertebrate abundances were 2.3 times higher at the moderately influenced site than within the goose colony, while abundances in upland habitat types were approximately equal. At the study sites, geese use the vegetated lowland habitats more than upland habitats, which have less vegetative cover [13]. Therefore, among study site variation in lowland invertebrate communities could be driven by goose-induced alteration of vegetation while upland habitats remain less affected.

We also found invertebrate family-specific habitat associations, which, if a habitat gradient is present could be contributing to driving these trends [59]. For example, the reduction in graminoid cover at both the moderately and highly goose-affected sites may be responsible for the increased abundances of Sciaridae and Chironomidae; families we found were both positively associated with low graminoid cover and goose pellet count, and were 3.7 and 1.9 times higher at the goose influenced sites than at the low goose influenced site. Similarly, Linyphiidae were associated with high fecal pellet counts and moss cover; both indices which were higher at and near the goose colony likely resulting in abundances of four to seven times higher at the goose influenced sites. A goose-driven habitat gradient and family-specific habitat preferences may therefore be further contributing to the variation in invertebrate abundances among sites.

Studies elsewhere have demonstrated that geese are able to alter invertebrate communities at the landscape scale. At a light goose stopover site in Delaware, [18] found invertebrate diversity, taxa richness, and abundance (particularly Chironomidae and Coleoptera) were significantly lower in habitats altered by geese compared to goose-excluded sites. Similarly, near a sub-Arctic light goose breeding site at La Pérouse Bay, [17] found spider abundance and species richness, and beetle abundance five, two, and seven times lower, respectively in significantly goose-altered habitat compared to unaltered sites. In both cases these trends were in part attributed to reductions in important habitat for invertebrates [17, 18] and geese indirectly ingesting invertebrates while grazing [18].

We suggest that at the largest scale (Southampton vs. Coats Island) invertebrate communities are benefiting from fecal deposition by geese, but at a smaller scale (within the colony vs. near the colony), goose-induced habitat alteration, particularly of graminoids and moss, reduces the suitability of habitat for invertebrates such as some Lycosid spiders and some Coleopterans. However, other more complex trophic interactions, which we were unable to test, may still be contributing to these trends. For example, habitat suitability for predatory invertebrates may be limited near the goose colony, potentially resulting in a trophic release and elevated abundances of low trophic level invertebrates. The high densities of shorebirds found nesting at the low goose influence site [13] could also be imposing top-down pressures on their prey, limiting invertebrate abundance. While these hypotheses require further in-depth study, perhaps with exclusion plots, enhanced abundances of invertebrates near the goose colony have the potential to benefit sympatric-nesting shorebirds.

The DNA metabarcoding results revealed specific prey items in shorebird diets, and the proportions of these prey families were broadly similar to their availability, as indicated by pitfall traps. On the breeding grounds, conventional dietary analysis of shorebirds indicates they consume a diversity of prey items [60, 61], but in some cases selectively target specific prey taxa [26]. Across the shorebird species studied here, using DNA metabarcoding, we detected 87 unique prey species but only a few families (Tipulidae, Chironomidae, Muscidae, Dytiscidae, and Carabidae) were consumed most frequently. These results corroborate previous gut analyses of breeding Red Phalarope and Semipalmated Sandpiper and provide the first quantitative analysis of White-rumped Sandpiper diet on the breeding grounds. In Barrow, Alaska, [62] found that Red Phalarope stomachs contained Chironomidae (50%) and Tipulidae larvae (33%), Carabidae (33%) and Plecoptera adults (10%), and low frequencies (<10%) of Muscidae pupae and Culicidae larvae. In Manitoba, adult Semipalmated Sandpiper frequently consumed Chironomidae larvae (60%), Arachnids (20%), and low (<10%) frequencies of Tipulidae, Muscidae, Dytiscidae, and Dolichopodid larvae, with some Dytiscidae adults [61]. Two adult White-rumped Sandpipers in the Canadian Arctic consumed large quantities of cranefly larvae (and one adult), three spiders, and one Carabidae larvae [60].

While a powerful and less invasive technique that requires less time and expertise in species identification than gut analysis, DNA metabarcoding does not provide a proportional consumption of each prey item, prey size, and is unable to differentiate among life stages of a single species [63]. Any differences in the importance of larval vs. adult Tipulidae, or their size, for example are missed. With these caveats in mind, DNA metabarcoding nevertheless provided us with an indication of relative importance of shorebird prey items during the breeding season and an opportunity to examine any variation in biomass or phenology of key prey items in greater detail.

We found significant inter-site variation in the peaks of biomass of specific shorebird prey items. Compared to the low goose site, Tipulidae biomasses and abundance peaks were substantially higher and appeared to occur slightly earlier within and near the goose colony. Chironomidae biomass was nearly 10 times greater at the goose colony and during one peak, abundance was 2.8 times higher at the moderate goose site than at the low goose site. Abundances of Muscidae were also similar among sites at the beginning of the season but at both high and moderate goose sites increased to nearly six times the abundance of the low goose site near the end of the summer. Similar to [8, 20] who found higher Chironomidae biomass and delayed Culicidae emergence in goose-altered ponds compared to unaltered sites, respectively, the trends we report are likely driven by elevated salinity in the goose colony, which may favor large salt-tolerant species and/or affect hydroperiod.

Regardless of the mechanisms, the reported goose-driven trends in prey biomass and phenology may have consequences for shorebirds. Elevated biomass of prey items near goose colonies could result in larger or higher quality eggs and even benefit chick growth rates of some species [25, 26]. Any changes in invertebrate phenology due to climate change [64], however, may result in a mismatch between timing of chick hatch and prey emergence potentially offsetting any positive effects associated with the goose colony for some [26, 30] but not all species [31, 65]. Furthermore, invertebrate emergence cycles vary significantly across broad-geographic scales [66], potentially obscuring any larger trends. We therefore recommend more site-specific studies and further research on goose-related effects on shorebird prey biomass and phenology to identify any potential population-level consequences (whether positive or negative).

## Conclusions

Over the last 60 years light goose populations have increased [33, 67] while many sympatric-nesting shorebird populations have declined [1, 2, 4]. Light goose-induced habitat alteration triggers trophic cascades, affecting invertebrate communities, their phenology, and their availability as prey for insectivorous birds. The effects of hyperabundant geese may not all be negative however. In areas with moderate grazing and habitat impacts, fecal pellet deposition may provide nutrients to support a more abundant and diverse invertebrate community. This enhanced prey base could support better adult body condition or higher rates of chick growth. However, the potential benefits of enhanced prey availability may also be offset by a reduced availability of preferred shorebird nesting habitat [13] and elevated risk of nest predation [12] within and near goose colonies. Combined, these complex interactions may be contributing, in part, to the spatial [68] and population-level declines documented in northern-breeding shorebirds [1, 3, 4, 69].

## Supporting information

S1 Table Variation in ground cover types within 1m^2^ areas situated at random points within six dominant habitat types at three study sites varying in goose influence (mean ± SE). (DOCX)

S2 Table Abundances (trap^-1^ day^-1^) of the dominant five invertebrate families among study sites varying in goose influence and habitat types (mean ± SE). (DOCX)
