# Supplementary material for: Habitat alteration and fecal deposition by geese alter tundra invertebrate communities: Implications for diets of sympatric birds
Source: PLoS One. 2022 Jul 1;17(7):e0269938. doi: 10.1371/journal.pone.0269938 (PMC9249211; doi:10.1371/journal.pone.0269938)
Supplement: S1 Table — (DOCX) [file pone.0269938.s001.docx]

S1 Table. Variation in ground cover types within 1m^2^ areas situated at random points within six dominant habitat types at three study sites varying in goose influence (mean ± SE).

| **Cover (%)** | **Goose influence** | **Dry Heath**  **n = 10** | **Gravel Ridge n = 10** | **Intertidal**  **n = 10** | **Moss Carpet n = 10** | **Sedge Meadow n = 10** | **Scrub Willow n = 10** |
| --- | --- | --- | --- | --- | --- | --- | --- |
| Rock | high | 13.75 ± 3.61 | 75.36 ± 4.61 | 32.50 ± 4.94 | 13.27 ± 4.46 | 0.62 ± 0.62 | 8.33 ± 4.99 |
|  | moderate | 19.26 ± 4.35 | 77.14 ± 11.01 | 37.92 ± 10.56 | 8.53 ± 3.76 | 1.00 ± 0.48 | 5.77 ± 3.06 |
|  | low | 22.71 ± 6.86 | 70.00 ± 14.39 | 69.17 ± 10.83 | 0.00 ± 0.00 | 0.16 ± 0.16 | 3.82 ± 1.52 |
| Lichen | high | 5.75 ± 0.83 | 3.21 ± 0.71 | 0.00 ± 0.00 | 0.38 ± 0.27 | 0.00 ± 0.00 | 1.13 ± 0.53 |
|  | moderate | 2.89 ± 0.91 | 0.71 ± 0.71 | 0.00 ± 0.00 | 0.29 ± 0.29 | 0.18 ± 0.18 | 0.85 ± 0.52 |
|  | low | 6.58 ± 1.47 | 2.14 ± 1.49 | 2.50 ± 1.71 | 0.00 ± 0.00 | 0.62 ± 0.37 | 0.41 ± 0.31 |
| Moss | high | 8.00 ± 2.19 | 0.00 ± 0.00 | 10.08 ± 3.49 | 54.81 ± 6.96 | 22.50 ± 5.26 | 14.53 ± 3.33 |
|  | moderate | 11.70 ± 5.14 | 7.86 ± 7.06 | 10.00 ± 6.60 | 45.41 ± 9.10 | 26.18 ± 7.32 | 15.62 ± 7.84 |
|  | low | 3.83 ± 1.88 | 12.86 ± 12.86 | 1.67 ± 1.67 | 60.83 ± 6.99 | 17.34 ± 3.37 | 9.41 ± 3.09 |
| Willow | high | 10.75 ± 2.62 | 2.86 ± 1.01 | 0.00 ± 0.00 | 2.96 ± 1.00 | 14.38 ± 6.71 | 31.6 ± 4.96 |
|  | moderate | 7.78 ± 2.49 | 0.71 ± 0.71 | 8.33 ± 6.61 | 2.94 ± 2.35 | 3.53 ± 1.34 | 12.08 ± 4.22 |
|  | low | 4.79 ± 1.98 | 0.00 ± 0.00 | 8.33 ± 8.33 | 5.42 ± 3.34 | 7.56 ± 2.53 | 15.76 ± 4.70 |
| Dryas sp. | GC | 30.25 ± 5.24 | 12.86 ± 4.80 | 0.42 ± 0.42 | 2.31 ± 1.60 | 0.00 ± 0.00 | 6.13 ± 3.12 |
|  | EBM | 27.04 ± 5.74 | 2.86 ± 2.86 | 0.00 ± 0.00 | 0.00 ± 0.00 | 1.18 ± 1.18 | 8.46 ± 5.97 |
|  | CI | 36.46 ± 5.50 | 10.00 ± 5.77 | 0.00 ± 0.00 | 0.00 ± 0.00 | 2.03 ± 0.89 | 10.29 ± 4.8 |
| Graminoid | high | 5.50 ± 2.23 | 0.00 ± 0.00 | 6.17 ± 3.92 | 6.15 ± 1.83 | 25.00 ± 6.48 | 3.67 ± 1.58 |
|  | moderate | 4.19 ± 1.45 | 3.57 ± 2.37 | 5.83 ± 2.81 | 13.24 ± 3.62 | 33.47 ± 6.67 | 10.46 ± 4.02 |
|  | low | 5.75 ± 1.31 | 0.71 ± 0.47 | 3.67 ± 2.40 | 21.92 ± 4.26 | 50.94 ± 5.33 | 12.12 ± 3.17 |
| Fecal Pellet Count | high | 4.40 ± 1.49 | 0.57 ± 0.29 | 10.75 ± 3.30 | 18.50 ± 5.31 | 22.88 ± 8.48 | 5.00 ± 0.76 |
|  | moderate | 7.67 ± 2.59 | 1.43 ± 1.27 | 4.25 ± 1.94 | 23.59 ± 11.51 | 17.88 ± 4.04 | 7.31 ± 3.02 |
|  | low | 1.42 ± 0.45 | 0.29 ± 0.29 | 0.00 ± 0.00 | 4.42 ± 1.14 | 2.19 ± 0.60 | 0.59 ± 0.23 |
