# Supplementary material for: Habitat alteration and fecal deposition by geese alter tundra invertebrate communities: Implications for diets of sympatric birds
Source: PLoS One. 2022 Jul 1;17(7):e0269938. doi: 10.1371/journal.pone.0269938 (PMC9249211; doi:10.1371/journal.pone.0269938)
Supplement: S2 Table — (DOCX) [file pone.0269938.s002.docx]

S2 Table. Abundances (trap^-1^ day^-1^) of the dominant five invertebrate families among study sites varying in goose influence and habitat types (mean ± SE).

| Taxon | Goose influence | Dry Heath | Gravel Ridge | Intertidal | Moss Carpet | Scrub Willow | Sedge Meadow |
| --- | --- | --- | --- | --- | --- | --- | --- |
| Chironomidae | high | 12.40 ± 4.32 | 7.33 ± 2.07 | 13.27 ± 2.85 | 9.05 ± 2.50 | 1.74 ± 0.29 | 1.68 ± 0.36 |
|  | moderate | 12.71 ± 3.66 | 7.51 ± 1.98 | 16.20 ± 3.70 | 8.83 ± 2.09 | 8.64 ± 3.40 | 19.34 ± 5.02 |
|  | low | 2.00 ± 0.30 | 3.40 ± 0.48 | 1.14 ± 0.29 | 0.00 ± 0.00 | 4.00 ± 0.83 | 4.06 ± 0.57 |
| Linyphiidae | high | 7.38 ± 1.19 | 1.42 ± 0.31 | 2.58 ± 0.48 | 10.42 ± 1.40 | 11.01 ± 2.95 | 1.17 ± 0.40 |
|  | moderate | 5.15 ± 1.48 | 1.21 ± 0.31 | 15.02 ± 1.17 | 8.52 ± 0.78 | 24.77 ± 5.23 | 15.46 ± 2.14 |
|  | low | 0.54 ± 0.11 | 0.30 ± 0.06 | 3.41 ± 1.06 | 3.28 ± 0.49 | 0.49 ± 0.07 | 1.40 ± 0.25 |
| Muscidae | high | 7.53 ± 1.84 | 2.82 ± 0.74 | 8.70 ± 2.10 | 7.03 ± 0.73 | 2.38 ± 0.45 | 3.94 ± 1.12 |
|  | moderate | 3.91 ± 0.92 | 2.60 ± 0.63 | 11.93 ± 3.48 | 5.42 ± 1.33 | 8.79± 1.77 | 9.00 ± 1.81 |
|  | low | 0.61 ± 0.10 | 0.60 ± 0.13 | 2.82 ± 0.64 | 5.18 ± 1.04 | 1.11 ± 0.22 | 0.37 ± 0.06 |
| Sciaridae | high | 8.58 ± 1.37 | 5.74 ± 1.45 | 3.90 ± 0.69 | 15.89 ± 2.86 | 0.85 ± 0.20 | 0.27 ± 0.07 |
|  | moderate | 5.44 ± 1.38 | 4.62 ± 0.72 | 105.64 ± 27.29 | 8.80 ± 2.36 | 21.31 ± 6.93 | 24.51 ± 7.83 |
|  | low | 1.56 ± 0.33 | 2.00 ± 0.27 | 4.53 ± 1.08 | 20.23 ± 3.56 | 1.28 ± 0.28 | 1.94 0.46 |
| Tipulidae | high | 4.35 ± 0.97 | 8.14 ± 2.46 | 0.94 ± 0.56 | 3.92 ± 1.41 | 4.89 ± 0.33 | 1.28 ± 0.33 |
|  | moderate | 6.49 ± 0.76 | 0.55 ± 0.08 | 0.31 ± 0.05 | 5.73 ± 1.89 | 8.28 ± 2.62 | 5.55 ± 1.26 |
|  | low | 0.14± 0.00 | 0.50 ± 0.00 | 0.21 ± 0.03 | 4.79 ± 1.01 | 4.89 ± 0.00 | 1.28 ± 0.00 |
