# Supplementary material for: Habitat alteration and fecal deposition by geese alter tundra invertebrate communities: Implications for diets of sympatric birds
Source: PLoS One. 2022 Jul 1;17(7):e0269938. doi: 10.1371/journal.pone.0269938 (PMC9249211; doi:10.1371/journal.pone.0269938)
Supplement: Attachment — Submitted filename: Response to Reviewers_PLoSONE.docx [file pone.0269938.s003.docx]

| **Comments from** | **Section** | **Line Number** | **Comment** | **Approach for Revision** | **Status** |
| --- | --- | --- | --- | --- | --- |
| Managing Editor | General | General | Please ensure that your manuscript meets PLOS ONE's style requirements, including those for file naming. The PLOS ONE style templates can be found.. | Revised as suggested | Addressed |
|  |  |  | We note that you have provided funding information that is not currently declared in your Funding Statement. However, funding information should not appear in the Acknowledgments section or other areas of your manuscript. We will only publish funding information present in the Funding Statement section of the online submission form.  Please remove any funding-related text from the manuscript and let us know how you would like to update your Funding Statement | We have removed the statement of funding in the Acknowledgements. The Acknowledgements should read:  *The authors would like to thank the community of Salliq (Coral Harbour), Nunavut, and the East Bay and Coats Island field crews for their assistance. We are especially grateful to Elyssa Cameron for sorting and identifying all invertebrates, Willow English for leading sample collection in 2017, and Sean Prosser for analysis of fecal samples. There is no conflict of interest between authors.*  The Funding Statement should read:  *Funding for this research was provided by the Arctic Goose Joint Venture, and scholarships provided to S.A.F. by the National Science and Engineering Research Council of Canada, W. Garfield Weston Foundation, and Ontario Graduate Scholarship. The funders had no role in study design, data collection and analysis, decision to publish, or preparation of the manuscript.* | Addressed |
|  |  |  | We note that you have stated that you will provide repository information for your data at acceptance. Should your manuscript be accepted for publication, we will hold it until you provide the relevant accession numbers or DOIs necessary to access your data. If you wish to make changes to your Data Availability statement, please describe these changes in your cover letter and we will update your Data Availability statement to reflect the information you provide. | We are currently preparing the data for submission to Dryad. In the interest of time we will provide the DOIs during the next submission/acceptance. | Addressed |
| Reviewer 1 | General | General | Goose-induced habitat alteration: the study design is based on the three study sites being differently affected by geese. The colony site being the most used, the site near the colony being moderately affected and the site far from the colony being less affected. At each of the sites, sampling was conducted in six habitats, which are also differently used by geese – and surely these habitats occur in different proportions at each site, very likely related to the activities of geese. I feel these differences between the sites supporting this goose-induced habitat alteration, as part of the base for the study design, are not very clearly presented in the manuscript (or at least I found this a little bit confusing, and reacted when reading in the first line of the discussion that the study identified “effects of goose-induced habitat alteration” on invertebrate community composition and abundance). | This is an excellent point. This is certainly very nuanced and we have attempted to respond to this in detail in several responses below. You are correct that the proportions of each habitat type are different at each site. This is in fact something that we have already investigated in great detail in a companion paper. Flemming, S.A., Nol, E., Kennedy, L.V., Smith, P.A., 2019. Hyperabundant herbivores limit habitat availability and influence nest site selection of Arctic‐breeding birds. Journal of Applied Ecology. https://doi.org/10.1111/1365-2664.13336. One of the findings in the other paper is that conclusions of this other paper is that the proportion of sedge meadow habitat (a habitat preferred by geese) increases with distance from goose colony. Throughout the manuscript, with focus on the methods section, we have tried to clarify the differences in sites and make reference to the already completed habitat work in our companion paper. We have further clarified some finer points in our responses below. We have greatly appreciate your comments and believe that have significantly improved the take home messages of this manuscript | Addressed |
|  |  |  | Site-level averages: I assume that the different habitats were represented at each site differently, but it is not clear if the averages at the site level were calculated taking these differences into account? For example, for the invertebrate samples, five pitfall traps were installed in each habitat at each site, but most likely the six habitat types are not equally represented at each site (especially under the assumption of habitat alteration by geese, at sites with different intensity of use by geese), so averages per site without taking into account the relative abundance of each habitat may lead to very different conclusions. | Thank you for picking up on this. You are absolutely correct that these differences would affect the "site-level" abundances and biomass. Since it was our intention to focus specifically on how invertebrates are affected by geese and then how shorebird diet is affected, when discussing the abundances we purposefully did not correct the numbers for total site as we viewed it as more important to compare abundances across habitat types at sites. However, when discussing biomass of invertebrate prey, we did correct for total site as we believed at the scale of a foraging shorebird this was more important. This was not clear in our original submission, but we have revised certain sections to make it explicit now. | Addressed |
|  | Abstract | 29 | Should this be “At sites and habitat types representing…”? The habitats are also differently used by geese, and otherwise the habitat types come as a surprise when you mention them for the first time in L35. | Changed as suggested | Addressed |
|  |  | 37 | “goose-influenced”? (same in L38) you use the hyphenated term later (L39) | Changed as suggested | Addressed |
|  |  | 45-46 | I see why you mention this here, but you did not assess if the effects on prey availability to shorebirds are outweighed by other factors, so this is not really a conclusion of your study. I wonder if a slightly different wording could help to make this clearer? (although I am maybe overthinking this – it just struck me the first time I read it. Feel free to ignore this comment if it does not make sense to you). | Changed to “Chick…growth. These benefits, however, might be outweighed by negative effects of goose-induced habitat alteration and predation pressure.” | Addressed |
|  | Introduction | 50 | Remove “of North America”? Repeats the beginning of the sentence. | Removed | Addressed |
|  |  | 81-83 | This sentence repeats what was said in L54-56. Leave out? | We very much appreciate your attention detail! We have left this sentence in as we feel it is important to first introduce the general subject and then in the later sentences describe how the effects are already described. | Addressed |
|  |  | 90-100 | Here I found a little bit confusing your description of your sites and the distance to the goose colony. If I understood it correctly, one of your sites is at the colony, another is some distance away and the last one is further away. You assume that the most pronounced goose-induced habitat alteration will be at the colony site, and decrease at the sites that are further away from the colony, right? You could maybe make this clearer in L93, for example, by referring specifically to the colony site when you mention the “site with the most pronounced goose-induced habitat alteration”? As well, in L97, you could directly refer to the sites with different intensity of goose disturbance instead of referring to distance (you use “site” rather than “distance” in your analyses, so I think this could be made clearer here). | These are excellent points and we thank you for asking for this clarification. Throughout the text, tables, and figures we have removed references to specific site names (except for when first mentioned) and replaced them with the degree of goose influence. For example, "Among study sites (F2, 280: 29.06, p < 0.001), graminoid cover was highest at the low goose influence site and lowest at the high goose site..." We also explicitly state in the methods "Previous studies at these sites have confirmed a gradient of goose effects at larger scales (12,13), thus, we refer to them here as high (goose colony), moderate (East Bay Mainland), and low (Coats Island) goose influence sites. " We hope this helps clarify. | Addressed |
|  | Methods | 110 | Would it be possible to include a map showing the locations of the three study sites? In addition, the map could include some information about each of the sites (e.g. habitat type composition, etc) | We have included a map of the study areas to help readers visualize the region. Given the scale of the region and the detailed mapping data required to produce this map we feel that including habitat type and composition is beyond the scope of this paper. We hope this satisfies the reviewer's concerns. | Addressed |
|  |  | 120 | I know the section includes “shorebird species” in the subheading name, but I feel this information would be better presented together with the shorebird diet later on. | We very much appreciate this suggestion. We have made the changes and agree that it has greatly improved the flow. | Addressed |
|  |  | 126 | How many locations were drawn randomly at each of the study sites? What was roughly the size of each site (within which the random points were located)? I understand that each of these locations (random sampling points) was characterized by its dominant habitat type, right? Would it be meaningful to then characterize each of the sites based on the relative abundance of the six habitat types? I think this would link better to the assumption of habitat alteration associated with the increased goose disturbance closer to the colony. | Thank you for catching this. We did not adequately explain in our original submission. We have included the sample sizes (5 surveys per habitat type per site per year) and areas in the manuscript. The areas of each of the study sites are as follows: High - 6 km2, Medium - 12 km2, 120, Low - 12 km2. | Addressed |
|  |  | 127 | Were the habitat surveys conducted once or over the three years of the study? | Good point. The habitat surveys were conducted in 2015 and 2016 in the years when invertebrate sampling took place. We have included these dates in the methods section. | Addressed |
|  |  | 137 | How does the placement of the pitfall traps relate to the habitat surveys described above (if at all)? | Excellent point and an oversight on our part. Habitat surveys were conducted at each of the pitfall trap sites and so invertebrate communities should reflect habitat variables in the immediate vicinity. We did not make this clear in our original submission but have clarified now. | Addressed |
|  |  | 137 | I guess the habitat types occurred to different extent at the three sites. Was then this taken into account to estimate prey availability at each site? | See more detailed comment above. As you suggest the biomass from the pitfall traps were multiplied by the proportions of each habitat type. We did not adequately describe this in the original submission but have changed it now. | Addressed |
|  |  | 156 | Comma after reference (37) | Revised as suggested | Addressed |
|  |  | 173 | Do you need to report here any details on data processing for the sequencing data? What software did you use (e.g. R packages)? | Our protocols followed those described by McClenaghan et al. 2019, and so we did not feel it was necessary to repeat the description of the lengthy process and thus refer the reader to this paper. We also cite R Version 10.0 later in the manuscript | Addressed |
|  |  | 177-178 | The differences between sampling points and sites are not part of your research questions (L85-89), although these analyses provide a nice confirmation of goose use of the sites and a description of the habitat types within each site. I wonder however, if these analyses should be presented as supplementary material? The same applies to the corresponding section in the results (L211-225). I feel the differences in ground cover types between habitats/sites are also less relevant to the study, unless you are looking at this as part of the goose habitat alteration process (in which case, this should be clarified, and probably presented as a separate question of your study?). In turn, the fecal pellet counts in the different habitats and sites provide very important confirmatory information for your choice of sites (see my comment below, to Table S1). Maybe this information could be highlighted better in the manuscript? | Thank you for this comment. It was originally our intention to make the links between habitat effects, invertebrate community, and shorebird prey availability. To make this clearer to the reader we have edited our goals sections to state this more explicitly. It now reads "Our objectives were to 1) confirm gradients of goose-induced habitat alteration were present at scales relevant to invertebrate communities, 2) examine any such effects of goose-induced habitat alteration on invertebrate communities, 3) determine which taxa of invertebrates are most frequently consumed by tundra-nesting shorebirds by characterizing their diet, and 4) identify any effects of geese on the biomass or timing of emergence of dominant shorebird prey items." We hope this satisfies the reviewer's concerns. Regarding the treatment of fecal pellets see response to comment on line 226 | Addressed |
|  |  | 180 | see my comment above about the average values per site. Following that, I am not sure it makes a lot of sense to include “site” here as a predictor variable (what does this variable really mean in this context?) | An excellent question and one we have debated for some time. The answer is of course very nuanced. Our hope was to structure the manuscript such that we first investigated habitat alterations specific to invertebrates, then differences in invertebrate abundances which are likely resulting from these habitat differences, and then consider how differences in biomass could affect shorebird diet. It is true that there are differences in the availability of habitats among study sites as you point out. When reporting the abundance estimates we chose not to estimate total abundance per site by multiplying by habitat availability for two reasons. 1) for this section we wanted to focus specifically on invertebrate communities and habitat-specific differences in abundance at a finer scale. 2) in the last section we wanted to focus specifically on how differences affect shorebird prey. Since we believe that total abundance is less important than site-specific biomass to shorebirds we chose to correct the biomass estimates not the abundance estimates. Throughout the manuscript we have revised sections to try to make this clear and have included one sentence stating this explicitly. We hope this satisfies your concerns. | Addressed |
|  |  | 195 | Please indicate here (or somewhere around L163?) how many samples you had per shorebird species. | We have included the sample sizes here for reference. | Addressed |
|  | Results | 211 | Weren’t habitat characteristics estimated within a 75m2 area surrounding the random sampling points (L130)? | Thank you for catching this. We have removed the following “within 1m^2^ areas at random points” which we hope will clarify | Addressed |
|  |  | 226 | What is really interesting from this table is the information on fecal pellet counts in the different habitats at the different sites. Shouldn’t this be presented in the main text as a figure, maybe as part of the site description? Were there differences between sites, and between the most used habitats (moss carpet and sedge meadow in GC and EBM)? What are the sample sizes for these comparisons? | We appreciate this comment. We have in fact already presented these data as part of a larger dataset as well as several similar sets of data such as vegetation height and lateral concealment in a companion paper from the same study sites (see above general response). We therefore felt that it was redundant to include such similar data and results in the main body of the text. However, since these data were a subset of the larger dataset they warranted being included in the supplementary material. We have attempted to draw the reader's attention to the companion paper and explicitly say that this assumption was already satisfied in reference to the companion paper. | Addressed |
|  |  | 231 | Maybe re-iterate here that this was over three summers? | We have clarified that this collection work took place “Over two summers at three study sites” | Addressed |
|  |  | 275 | I found Figure 1 a little difficult to follow. I think the comparisons between different habitats and sites would be clearer by presenting bars (with the corresponding errors) for each habitat at each site (instead of dots). I wonder also about the boxplots. They are useful to show differences between sites – but were these weighted by the proportion of the different habitat types at each site? Also, it would be good to follow the same order of presentation as in Tables 1 and 2 (first Chironomidae, then Linyphiidae, etc) | We appreciate your suggestions on this figure and have considered them all in painstaking detail. We have visualized the figure in several different ways including the one you suggested. While we do admit that the various lines do make the figure somewhat more challenging to read, viewing the trends by habitat and family results in 30 individual plots which present their own challenges with respect to space and interpretation. We find that the figure we present shows variations in site and among habitat types in the most parsimonious way possible. Respectfully we also feel that barplots are rarely the best way to visualize scientific data and have therefore chosen to present the data as a dot plot still. Regarding the weighting of the site averages and use of boxplots, please see our general response and response to comment on line 180 which explains our rationale in more detail. Lastly, you are absolutely right the same order should be followed as in table 1 and 2. We have rectified this and hope this satisfies your concerns. | Addressed |
|  |  | 308 | The resolution of Figure 2 is really poor! | Thank you for pointing this out. We have redone both figures 2 and 5 to make sure they are of higher resolution and quality. | Addressed |
|  |  | 334 | See comment above – please provide sample sizes in the text when you describe the sampling for shorebird diets | See above response | Addressed |
|  |  | 353 | Are the lines between the points really needed? | Thank for you your comment. We agree with your comment and have removed the lines between points. | Addressed |
|  | Discussion | 365 | From the pellet counts (presented in Table S1) it is not very clear that East Bay Mainland had intermediate goose pressure? | Good catch! Our companion paper delves into habitat-related changes at broader scales in much much more detail using a larger data set (of which this is a subset of). In this companion paper there are very clear differences in lateral concealment, vegetation height, and availability of certain habitat types (as you rightly pointed out). To support our assertion we have provided references to this paper further, explicitly stated the link, and softened the language to ensure that we do not mislead the reader. | Addressed |
|  |  | 370-375 | I think it would be very helpful to have this presented as a figure – as it is presented now, this information is buried in a supplementary table. | See response to comment on line 226 | Addressed |
|  |  | 381 | “at sites at different distances from the goose colony”? | Good catch. Thank you for helping us clarify. We have revised. | Addressed |
|  |  | 384 | This is the first time you refer to lowland vs upland habitats. If this is important, it should be introduced earlier. | Thank you for pointing this out. We have referenced the lowland and upland habitat types explicitly in the methods section now where we describe the habitats. | Addressed |
| Reviewer 2 | Discussion | General | Would be good to Indicate the name of trap. Is it Barber H. Traps for cave-inhabiting insects // J. Elisha Mitchell Sci Soc.- 1931.- V.46.- P.259-266.? | While it is interesting to note the first description of these traps we have described our very simple pitfall traps and hope that this description is sufficient. | Addressed |
